# Statistical and methodological problems with concreteness and other semantic variables: A list memory experiment case study

**DOI:** 10.3758/s13428-017-0938-y

**Published:** 2017-07-13

**Authors:** Lewis Pollock

**Affiliations:** University College London, London, UK

**Keywords:** Concreteness, Semantic variables, List memory, Methodology

## Abstract

The purpose of this article is to highlight problems with a range of semantic psycholinguistic variables (concreteness, imageability, individual modality norms, and emotional valence) and to provide a way of avoiding these problems. Focusing on concreteness, I show that for a large class of words in the Brysbaert, Warriner, and Kuperman (Behavior Research Methods 46: 904–911, 2013) concreteness norms, the mean concreteness values do not reflect the judgments that actual participants made. This problem applies to nearly every word in the middle of the concreteness scale. Using list memory experiments as a case study, I show that many of the “abstract” stimuli in concreteness experiments are not unequivocally abstract. Instead, they are simply those words about which participants tend to disagree. I report three replications of list memory experiments in which the contrast between concrete and abstract stimuli was maximized, so that the mean concreteness values were accurate reflections of participants’ judgments. The first two experiments did not produce a concreteness effect. After I introduced an additional control, the third experiment did produce a concreteness effect. The article closes with a discussion of the implications of these results, as well as a consideration of variables other than concreteness. The sensorimotor experience variables (imageability and individual modality norms) show the same distribution as concreteness. The distribution of emotional valence scores is healthier, but variability in ratings takes on a special significance for this measure because of how the scale is constructed. I recommend that researchers using these variables keep the standard deviations of the ratings of their stimuli as low as possible.

Word concreteness has become one of the most studied variables in the psycholinguistic literature. Since Paivio, Yuille, and Madigan (1968) published one of the first large-scale databases of word concreteness norms, “concreteness effects” have emerged in a variety of investigations of various cognitive processes, and a range of theories have been proposed in an attempt to explain these effects. Independent teams of researchers operating over a period of decades have repeatedly shown that concrete words show a processing advantage over abstract words in certain experimental paradigms. For example, concrete words are easier to remember than abstract words (Allen & Hulme, 2006; Miller & Roodenrys, 2009; Romani, McAlpine, & Martin, 2008; Walker & Hulme, 1999), are easier to make associations with (de Groot, 1989), and are more easily and more thoroughly defined in dictionary definition tasks (Sadoski, Kealy, Goetz, & Paivio, 1997). Historically, it was claimed that concrete words are responded to more quickly than abstract words in lexical decision tasks (Bleasdale, 1987; James, 1975; Kroll & Merves, 1985), although more recent experiments have shown no difference (Brysbaert, Stevens, Mandera, & Keuleers, 2016), or even that abstract words might have an advantage after various other variables have been accounted for (Kousta, Vigliocco, Vinson, Andrews, & Del Campo, 2011). However, even an abstractness advantage in lexical decision points to the utility of word concreteness as a psycholinguistic variable.

Brain-imaging techniques have also been employed to determine whether the neural systems underpinning concrete words and abstract words are distinct (Binder, Westbury, McKiernan, Possing, & Medler, 2005; Dhond, Witzel, Dale, & Halgren, 2007; Kounios & Holcomb, 1994; Pexman, Hargreaves, Edwards, Henry, & Goodyear, 2007; Sabsevitz, Medler, Seidenberg, & Binder, 2005). The general consensus from these brain-imaging studies is that there is evidence of a neuroanatomical difference in the processing of concrete versus abstract words.

Psychologists are clearly heavily invested in the investigation of word concreteness, and for good reasons. If there are properties that define a cognitively relevant ontology of concepts, concreteness seems like a good candidate: Something about what constitutes the concept of “elephants” (highly concrete) is probably different from what constitutes the concept of “paradoxes” (highly abstract). However, in this article I will highlight a problem with the concreteness measure, based on a simple statistical summary of the Brysbaert, Warriner, and Kuperman (2013) concreteness norms. I report three replication experiments that together suggest that this problem is not fatal to concreteness research, but also that it should be acknowledged when researchers design their stimuli. I also show that the same problem applies to other variables in semantic databases, such as imageability (Cortese & Fugett, 2004; Schock, Cortese, & Khanna, 2012) and individual modality norms (Lynott & Connell, 2012).

## Word concreteness

A word’s concreteness rating is derived by asking a group of participants to rate that word for concreteness on a Likert scale. A low score indicates that a word is highly “abstract,” whereas a high rating indicates that a word is highly “concrete.” The mean value of all participants’ ratings is taken to be an approximation of a word’s position on an abstract-concrete continuum. I will now develop some theoretical concerns about the validity of traditional concreteness norms before turning to a statistical analysis of the Brysbaert et al. (2013) database. Consider the job a participant is being asked to do when she is told to rate a word between, say, 1 and 5 on a scale of concreteness. She is told that “concrete words are experienced by the senses,” whereas abstract words are not (Paivio et al., 1968). For some words, the interpretation of traditional concreteness norming instructions is relatively straightforward. A participant who is presented with the word “apple” is likely to have seen, touched, smelled, and tasted apples throughout the course of their life, and will unproblematically assign “apple” a high concreteness rating. Similarly, a participant that is presented with the word “serendipity” is likely to reason that since serendipity is a loose association between some coincidental, nonspecified events, and is not something that affords direct sensory experience, the word “serendipity” should be assigned a low concreteness rating. However, what are the properties that a word/concept should have in order for it to be assigned a mid-scale rating? It is difficult to formulate a coherent approach to this task: Can an entity or idea be “half-seen” or “half-touched”? What does it mean to have intermediate sensory experience of an entity or idea? That is to ask: What is a participant telling us about a word when they rate it a 3 out of 5? They could mean any one of the following:

1. Adding up all of my sensory experience of this object across all five of the sensory modalities, I realize that I have seen and heard it, but never touched, smelled, or tasted it. So I suppose I’ll rate it a 3.
2. One interpretation of this word brings to mind something that cannot be directly experienced, whereas a different interpretation of this word brings to mind something that can be directly experienced. So I suppose I’ll rate it a 3.
3. Sometimes I associate sensory experience with this word, but sometimes I don’t. So I suppose I’ll rate it a 3.

It is certainly possible to imagine more potential approaches, and there is no empirical basis for selecting one of these approaches over another. Furthermore, it is likely that different participants will generate different interpretations for many of the words in any list of words to be normed. When a participant sees the letter string < deed > presented in isolation, there is no way that a researcher can control for the fact that half of the participants may interpret < deed > as referring to a document associated with proof of property ownership (high concreteness value?), and the other half may interpret it as referring to some unspecified action, perhaps involving some element of heroism (low concreteness value?). Consequently, for a number of words it is just not clear what word/concept the mean concreteness rating is supposed to reflect.

This point on its own might be enough to motivate the avoidance of words with a mean value in the middle of a concreteness–abstractness scale. Given that it is not clear what it is that participants are even telling us when they rate a word a 3, we might also wonder how often participants actually use values from the middle of the concreteness scale when making their judgments. Recently, Brysbaert et al. (2013) provided a concreteness norm database of 40,000 English words, which dwarfs the previously popular MRC database used in most studies (Coltheart, 1981). This new, larger database allows a statistical analysis of the distributions of concreteness norms across a much larger section of the English lexicon. I now present this analysis and use it to develop the concerns raised in this section.

## Brysbaert et al. (2013) concreteness norms

Brysbaert et al. (2013) collected a new set of concreteness norms for 40,000 English words. Groups of approximately 25 participants rated subsets of the whole list of 40,000 words on a concreteness scale of 1 (*very abstract*) to 5 (*very concrete*). The participants (*n* = 4,237) came from a range of ages, with approximately one third between 17 and 25 years old, and two thirds between 26 and 65. The mean value of a group of participants’ judgments about the concreteness of a stimulus word was assumed to be a useful approximation of that word’s position on a hypothesized concrete–abstract continuum. I shall now argue that this is not necessarily the case. The standard deviation of a dataset is a measure of the average distance between all data points in that dataset and the mean value of all data points in the dataset. If every participant rates a word as a 1 (*highly abstract*), then that word’s concreteness rating will have a standard deviation of 0. However, if half of the participants rated a word as a 1, but the other half rated the word as a 5 (*highly concrete*), that word would have a mean concreteness rating of 3 but a standard deviation of 2. In Likert scale norming tasks, the standard deviation of a set of ratings is therefore a blunt index of the extent to which participants agreed with each other about how a word should be rated.

**Fig. 1:**
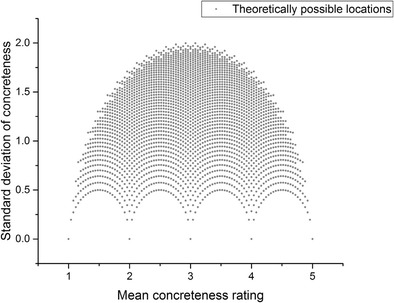
Theoretically possible locations for words rated between 1 and 5 by 25 different participants

If a dataset contains 25 numbers (in our case, 25 individual concreteness judgments), all of which are integers between 1 and 5, then there are a finite number of possible combinations of means and standard deviations for that dataset. Figure 1 below plots all of these possible combinations:

Note how, at the extreme ends of the *x*-axis, only a standard deviation of 0 is possible, because for a mean value to be 1 or 5, all 25 participants must have rated a word as 1 or 5, respectively. However, in the middle of the scale the disagreement that is theoretically possible increases, reaching a peak at mean value ~3, standard deviation ~2. Crucially, it is still theoretically possible for a data point to occur with a mean value located in the middle of the scale, but with a relatively low standard deviation. That is, it is still clearly theoretically possible for participants to more or less consistently agree that a word is of intermediate concreteness.

**Fig. 2:**
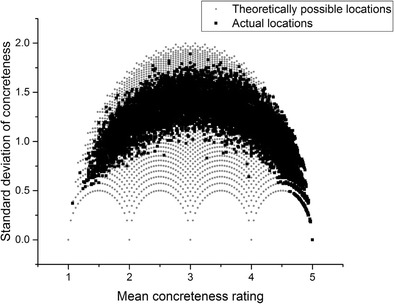
Theoretical versus actual locations

Now, consider Fig. 2, which plots the *actual* mean concreteness value and the standard deviation of every noun in the Brysbaert et al. (2013) concreteness norm dataset (*n* = 14,592) over the top of the *theoretically possible* combinations depicted in Fig. 1.

The pattern is striking. At the extreme concrete end of the scale, many items have high concreteness ratings and relatively low standard deviations, indicating that participants more or less agreed in their judgments about how to rate these words. At the extreme abstract end of the scale, there are likewise words with low concreteness ratings and relatively low standard deviations, although not to the same extent as at the extreme concrete end. However, in the middle of the scale there is an obvious rise in the standard deviation. Only a handful of words have a mean value near 3 and a standard deviation even slightly below 1. Indeed, a large class of words have a standard deviation well over 1, ranging from mean values of 1.5 to 4.5.

This indicates that for a great number of items, participants were not agreeing in their judgments of how concrete a stimulus word was. At mean values of 2 and 4 there are many cases of standard deviations above 1. Remember that ratings on this scale can only take integer values between 1 and 5. This means that for many of the words with a mean value of 2 or 4, some participants must have judged these words as belonging at the opposite end of the concreteness scale from the position where the mean value suggests the word belongs. This phenomenon is problematic for the assumption that concreteness should be treated as a continuous variable. This is because in a vast number of cases, participants’ judgments tended not to be continuous; instead, they tended to be binary: Participants were using values of 1, 2, 4, and 5 in producing these concreteness norms, and avoided using 3. Furthermore, in many cases participants were judging a word as a 1 (*totally abstract*), whereas others were judging that same word as a 4 (*somewhat concrete*).

Given these methodological issues, it might seem surprising that concreteness effects are so widely reported. If measurements for a large section of the hypothesized concreteness spectrum are actually procedural artifacts, it is then unclear what phenomenon it is that concreteness effects are actually indexing. One potential explanation is that generally, when investigating the effect of a variable, researchers try to choose stimuli that maximize a change in this variable, in order to generate the maximum possible effect. It is therefore possible that empirical concreteness research might not suffer too badly from the problem of binary disagreements concerning midscale items, because researchers will have aimed to pick stimuli from the extreme ends of the scale, and these polar items are less subject to disagreement.

However, if it turns out that many experimental stimuli do suffer from the disagreement phenomenon, this poses an explanatory problem concerning the evidence in favor of processing differences between abstract and concrete items. The typical finding is that there are processing advantages for concrete items relative to abstract items, and the typical explanation of this finding is that concrete and abstract items have different neurologically instantiated formats and/or structural relationships. If a significant number of the stimuli included in an abstract or concrete experimental condition actually come from the middle of the concreteness scale, then the typical claim that there are processing differences between concrete and abstract items is no longer supported by the data. This is because words from the middle of the scale *must* have high standard deviations. This means that only half of the participants who produced the concreteness measure for that word judged it to be abstract, and the other half judged it to be concrete. Therefore, there are no empirical grounds for calling these words “concrete” or “abstract” in the first place.

## Stimuli in concreteness experiments: A case study of list memory paradigms

In this section I plot the stimuli featured in four list memory experimental studies against the entire Brysbaert et al. (2013) database. These studies are Allen and Hulme (2006), Walker and Hulme (1999), Romani et al. (2008), and Miller and Roodenrys (2009). We should note a few things. First, although the replication experiments that I report below feature noun stimuli, and most studies under discussion here also featured nouns, occasionally their stimulus sets featured other word classes alongside nouns. In the case of Allen and Hulme, many of the stimuli in the abstract condition were not nominal. Therefore, to display the maximum number of stimuli for all experiments, I have plotted the entire Brysbaert et al. (2013) database (*n* = 40,000) instead of just the nominal subsection of it. Not all of the stimuli featured in all experiments appeared in the Brysbaert et al. norms, and these stimuli have been omitted from the analysis. Second, the pattern of means and standard deviations is absolutely unchanged when we compare the entire Brysbaert et al. database with the noun subsection of it.

**Fig. 3:**
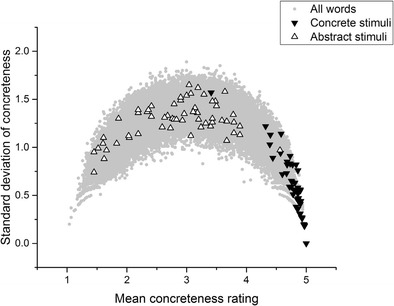
Romani et al. (2008) stimuli

Now, consider Fig. 3. The stimuli featured in Romani et al. (2008) best exemplify the problem, although the intention here is not to single out Romani et al. or any of the other authors under discussion for criticism. The analysis I present here would have been almost impossible to carry out at the time that these experiments were conducted, given that the Brysbaert et al. concreteness database was only published in 2013. In brief, the problem is that the concrete words tend to have low standard deviations, whereas the abstract stimuli tend to have high standard deviations and to be drawn from the middle of the scale, rather than the unequivocally abstract part of the scale. This is potentially problematic for the validity of Romani et al.’s conclusions regarding concreteness effects, because many of the stimuli that made up their abstract stimuli were not unequivocally abstract. For the standard deviations of many of the “abstract” stimuli to be as high as they are—in many cases, well above 1—many participants must have been judging those words to be concrete during the Brysbaert et al. (2013) norming process. Some of the abstract stimuli have standard deviations approaching the theoretical maximum of 2, indicating maximum disagreement among participants about whether that word is concrete or abstract. To reiterate: Participants could only apply integer values in making their judgments. Therefore, even if a word has a mean concreteness rating of approximately 2, but also a standard deviation of the rating above 1, that means that some participants must have been crossing scale halves in making their judgments. Ultimately, it is not clear what comparison is actually being made here. The concrete stimulus lists were more or less unproblematically concrete. However, the abstract stimulus lists contained words drawn from nearly the entire length of the concreteness scale, and also tended to feature words that participants disagreed about how to rate.

**Fig. 4:**
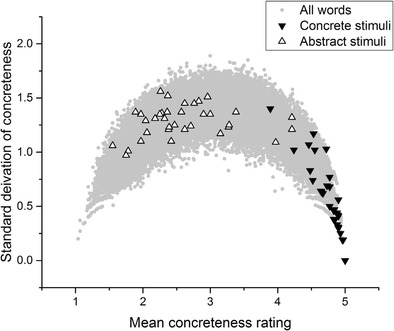
Allen and Hulme (2006) stimuli

Figure 4 depicts the abstract and concrete stimuli featured in Allen and Hulme (2006). Again, many “abstract” stimuli here have standard deviations well above 1, indicating that people disagreed about whether the words were abstract in the first place. The range of mean ratings of concreteness for the abstract condition is also clearly much higher than in the concrete condition. Once again, a relatively homogeneous group of concrete words has been compared to a heterogeneous group of words about which participants tended to disagree.

**Fig. 5:**
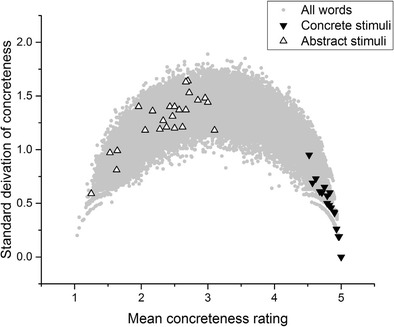
Miller and Roodenrys (2009) stimuli

Figure 5 plots the stimuli featured in Miller and Roodenrys (2009). Again, there is a marked difference in standard deviations between the concrete and the abstract stimuli. Furthermore, the standard deviations of the abstract stimuli are so high (well above 1 in the majority of cases) that the mean value does not reflect the judgments that participants were actually making.

**Fig. 6:**
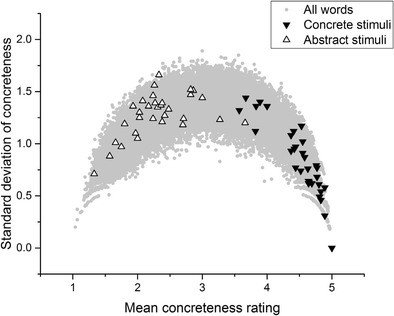
Walker and Hulme (1999) stimuli

Finally, consider Fig. 6, which depicts the stimuli featured in Walker and Hulme (1999). The midscale criticism applies least to this set of stimuli, although it is still clearly the case that the concrete stimuli tended to have lower standard deviations than the abstract stimuli. The reasons for this have already been expounded. The upshot is that a skeptic could reasonably argue that these experiments do not actually provide evidence for concreteness effects. The reason is that the comparison being made was meant to be between concrete and abstract items, but the comparison that was actually made was between concrete items, on the one hand, and a group of stimuli about which participants disagree, on the other. It *could* be the case that words that engender disagreement are those that are hard to remember, and that this explains processing differences that have previously been attributed to concreteness/abstractness. The experiments that I report below were designed to test this possibility.

Before moving on to a report of these replication attempts, I wish to point out that list memory paradigms are not a special case when it comes to the properties of “abstract” stimuli. Table 1 presents a number of experimental concreteness studies from a wide variety of paradigms, as well as a summary of the concreteness values and standard deviations of the stimuli featured in their experiments. The abstract–midscale stimulus pattern applies to every single experiment.

**Table 1:** Concreteness statistics in various experimental paradigms

Article	Type of Data	Experimental Paradigm	Concrete		Abstract	
			Mean Concreteness	Mean *SD*	Mean Concreteness	Mean *SD*
Kroll & Merves (1985)	Behavioral	Lexical decision	4.55	0.74	2.17	1.22
de Groot (1989)	Behavioral	Word association	4.66	0.6	2.36	1.24
Paivio et al. (1994)	Behavioral	Recall	4.83	0.47	2.29	1.28
Gee et al. (1999)	Behavioral	Recall	4.73	0.57	3	1.33
Binder, Nelson, & Krawczyk (2005)	fMRI	Lexical decision	4.76	0.52	2.34	1.23
Crutch & Warrington (2005)	Patient population	Word matching	4.83	0.46	3.53	1.18
Sabsevitz et al. (2005)	fMRI	Semantic judgment	4.86	0.45	2.58	1.31
ter Doest & Semin (2005)	Behavioral	Recall	4.72	0.57	2.45	1.26
Lee & Federmeier (2008)	EEG	Semantic judgment	4.41	0.88	2.27	1.24
Huang et al. (2010)	EEG	Semantic judgment	3.82	1.17	2.53	1.21
Skipper-Kallal, Mirman, & Olson (2015)	fMRI	Deep thought	4.44	0.81	2.38	1.22
Jager & Cleland (2016)	Behavioral	Lexical decision	4.62	0.64	3.29	1.19

Once again, I stress that none of the analysis presented here is intended as a specific criticism of any of these studies. These studies were chosen simply because they reflect a range of experimental paradigms (lexical decision, recall, semantic judgment, word association, and picture–word matching), data types (behavioral, fMRI, electroencephalography [EEG]), and include both neurotypical and patient populations. They also, laudably, included their stimulus sets in their experimental reports, although it is important to note that for Sabsevitz et al. (2005) and Lee and Federmeier (2008) only samples of the stimuli were available. For every study but one listed in Table 1, the mean standard deviation of the stimuli in the concrete condition was below 1, whereas the mean standard deviation of the stimuli in the abstract condition was above 1. The only exception is Huang, Lee, and Federmeier (2010), in which the standard deviations for both stimulus sets were relatively high. Looking at the distributions displayed above in Figs. 2, 3, 4, 5 and 6, it is clear that the only way these statistics could be obtained is if the midscale disagreement problem applied to all of the abstract stimulus sets of the experiments depicted in the table. I now turn to a report of three new list memory replication experiments in which I attempted to control for the problems that I have outlined so far.

## Experiment 1

**Fig. 7:**
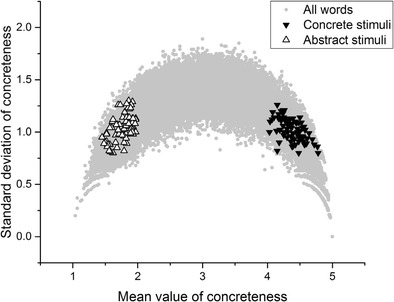
Concrete and abstract stimuli featured in Experiment 1

The purpose of this experiment was to replicate an experiment reported in Romani et al. (2008) while controlling for the potentially problematic confound between the mean value of a concreteness rating and the standard deviation of that rating. Romani et al. presented participants with lists of words and asked them to recall words from that list immediately after the presentation of the last word of the list. Romani et al. reported that participants were better at recalling lists of words that consisted entirely of concrete words than at recalling lists that consisted entirely of abstract words. Experiment 1 here investigated the reliability of this concreteness effect when the standard deviations of the concreteness value of the words across lists was controlled, while also directly manipulating words’ standard deviation in order to ascertain whether the standard deviation itself has a significant effect on task performance. Figure 7 plots the mean concreteness values and standard deviations of concreteness of the concrete and abstract stimuli used in the present experiment in the same way that the stimuli used in previous experiments were plotted in the previous section.

We can see that the contrast in concreteness between conditions is maximized and that the difference in the standard deviations of concreteness ratings is controlled. Of interest is whether the concreteness effect would still occurs when these new controls were enforced.

The specific Romani et al. (2008) experiment replicated here is Experiment 3 B, which is a free-recall task in which participants simply try to recall any word from the list that they can, regardless of order. Romani et al. reported that concreteness effects are stronger in free-recall than in serial-recall tasks, so a free-recall task provides the most robust test of the concreteness effect. An additional two experimental conditions were added: agreement and disagreement conditions. Words in the *agreement* condition were taken from the middle of the scale and had relatively low standard deviations, and words in the *disagreement* condition were taken from the middle of the scale and had relatively high standard deviations. Summary psycholinguistic statistics for all conditions are given in the Materials section below. Three comparisons were of interest: concrete versus abstract, concrete versus disagreement, and concrete versus agreement. In this way, the importance of the midscale problem outlined in the section above can be assessed.

## Method

### Participants

Originally, 60 native speakers of English with no reported neurological disorders were recruited from the University College London SONA psychology pool. Of these, 50 completed the experiment (the other ten either did not turn up or canceled their session). All participants were either awarded course credit or paid £6 for their time.

### Materials

Forty lists, each containing eight words, were generated. There were four experimental conditions, each of which comprised ten lists. The stimuli were controlled for the following psycholinguistic variables: standard deviation of concreteness, frequency, age of acquisition, number of phonemes, number of letters, and number of syllables. Table 2 contains the mean values (with standard deviations in parentheses) of each of these variables for each condition.

**Table 2:** Stimulus properties

Condition	Mean Concreteness	*SD* Concreteness	AoA	Zipf Frequency	L Phon	Length	*N* Syll
Concrete	4.38 (0.17)	1.02 (0.11)	10.45 (2.05)	3.34 (0.79)	5.59 (0.94)	6.93 (1.06)	2.00
Abstract	1.78 (0.14)	1.04 (0.12)	10.58 (2.09)	3.38 (0.83)	5.56 (0.93)	6.84 (1.17)	2.00
Agree	3.17 (0.7)	1.08 (0.07)	10.09 (1.9)	3.15 (0.85)	5.63 (1.03)	6.93 (1.21)	2.00
Disagree	3.1 (0.36)	1.65 (0.05)	10.23 (2.04)	3.13 (0.81)	5.76 (1.10)	6.9 (1.32)	2.00

Mean concreteness: Mean concreteness rating; *SD* concreteness: The mean standard deviation of the concreteness ratings; AoA: Age of acquisition; Zipf frequency: Word frequency in Zipf units; L Phon: Length of word in phonemes; Length: Length of word in letters; *N* Syll: Number of syllables

Psycholinguistic variable information was gathered from Brysbaert et al. (2013), Kuperman, Stadthagen-Gonzalez, and Brysbaert (2012), and the English Lexicon Project (Balota et al., 2007). The stimulus sets were created using MATCH (van Casteren & Davis, 2007). The four conditions were concrete, abstract, agreement, and disagreement. *Concrete* lists contained words that had mean values between 4 and 5 on the Brysbaert et al. (2013) concreteness scale. *Abstract* lists contained words that had mean values between 1 and 2 on the Brysbaert (2013) concreteness scale. The *agreement* and *disagreement* lists contained words that had mean values between 2.5 and 3.5 on the Brysbaert et al. (2013) concreteness scale. The concrete, abstract, and agreement lists were constructed such that the standard deviations of the concreteness ratings of the words in those lists were similar, whereas the disagreement condition was formed exclusively of stimuli with high standard deviations. Table 3 contains a sample list from each condition, and full lists of the stimuli featured in all experiments reported in this study are included in the Appendix.

**Table 3:** Sample stimulus lists

Condition	Word 1	Word 2	Word 3	Word 4	Word 5	Word 6	Word 7	Word 8
Concrete	Beaker	Clinic	Tango	Clothing	Amber	Jackal	Roulette	Survey
Abstract	Desire	Mystique	Intent	Vantage	Glory	Nuance	Unease	Motive
Agree	Diesel	Roughhouse	Attempt	Whiner	Viewpoint	Freshness	Stampede	Leader
Disagree	Slipstream	Audit	Poorhouse	Minute	Rival	Tribune	Abyss	Spectrum

### Procedure

The experimenter read all of the words from a list one after the other. There was a 2-s pause between consecutive words being read out. The order of the lists and the order of the words within each list were randomized for each participant. After the experimenter had finished reading out a list, the participant spoke out loud any and all words that he or she could remember from that list. The experimenter recorded every word that the participant spoke. Because this was a free-recall task, the order in which the participants recalled the words did not matter. Participants were not penalized for making errors or substitutions, or for saying a word that had not actually been in the list. The experiment lasted approximately 35 min.

## Results

Table 4 summarizes the mean numbers of words remembered (and standard deviations) by condition.

**Table 4:** Mean words recalled by condition for Experiment 1

Condition	Mean Words Recalled (*SD*)	Mean Percentage Recalled
Concrete	4.67 (1.35)	58.4%
Abstract	4.48 (1.24)	56%
Disagree	4.38 (1.28)	54.6%
Agree	4.45 (1.35)	55.6%

The results were analyzed with a mixed-effects model in R using the lme4 package (Bates, Mächler, Bolker, & Walker, 2015). The lmertest package was used in order to obtain *p* values for the comparisons of interest via Satterthwaite approximation (Kuznetsova, Brockhoff, & Christensen, 2015). The mixed-effects model examined the fixed effect of experimental condition on the number of words remembered per trial, with subjects and items being treated as random effects with varying intercepts.

The statistical contrasts were the abstract, disagreement, and agreement conditions versus the concrete condition. That is, a treatment contrast with the concrete condition representing the baseline condition. Table 5 displays the results of this analysis.

**Table 5:** Summary of mixed-effects model for Experiment 1

Fixed Effects	Effect Estimate	Error	*df*	*t*	*p*	Lower 95%CI for Effect	Higher 95%CI for Effect
Abstract	–.19	–.12	39.25	–1.56	.13	–.43	.05
Agree	–.22	–.12	39.25	–1.79	.08	–.46	.03
Disagree	–.29	–.12	39.25	–2.42	.02	–.54	–.05

Because three nonindependent hypothesis tests were run on the same data, a Bonferroni correction was applied. Assuming a conventional alpha level of .05, the corrected alpha level was therefore .05/3 = .017. The concrete–abstract contrast was not statistically significant (*p* = .13). Therefore, there was no evidence for an advantage for concrete over abstract word lists, contrary to the findings of Romani et al. (2008), Walker and Hulme (1999), Allen and Hulme (2006), and Miller and Roodenrys (2009). None of the other contrasts were statistically significant, either, at the Bonferroni-corrected alpha level (concrete vs. agreement, *p* = .08; concrete vs. disagreement, *p* = .02). There was therefore no evidence words from the middle of the concreteness scale are simply harder to remember than words from the extreme concrete end of the scale, and there was no evidence that words with high standard deviations in rating are harder to remember than words from the extreme concrete end of the scale. However, a reviewer raised the important point that Experiment 1 suffered from a lack of power, because there were only ten items per condition. This could be the reason that no statistically significant results were obtained.

To account for this possibility, the data were reanalyzed using a Bayesian model comparison analysis in the BayesFactor package for R (Morey, Rouder, & Jamil, 2015) with the default settings and priors. If the results of the frequentist analysis presented in the preceding paragraphs were due to low power, then the Bayes factors produced by this analysis are likely to be between 1/3 and 3, which would indicate that the data do not decide the issue either way.

Kruschke (2011, p. 310) argued that the Bayes factor generated from a model comparison analysis of an experimental design with multiple conditions may be misleading for various reasons. Therefore, the total results dataset of Experiment 1 was partitioned into three smaller datasets that reflected the pairwise comparisons of interest between the conditions: one concrete–abstract comparison, one concrete–agree comparison, and one concrete–disagree comparison. In every case, a model including a parameter for the fixed effect of condition was compared to a null model that featured only subjects and items as random effects. The resulting Bayes factors for each comparison were concrete versus abstract, 0.32; concrete versus agree, 0.38; concrete versus disagree, 0.66. For the concrete–abstract comparison, there is marginal evidence in favor of a null effect (BF = 0.32). For the other two comparisons, the Bayes factor indicates that the data do not decide between the null or alternative models. Taken together with the frequentist analysis presented previously (all *p* values above the threshold for statistical significance), these results suggest no difference in recall between the concrete and abstract conditions. However, the evidence for a null difference in the other comparisons is inconclusive.

Before moving on to the second replication experiment, it is important to note a shortcoming of Experiment 1 that may have affected the results. The standard deviations of the concreteness ratings of both the concrete and abstract stimuli were relatively high: above 1, in many cases. It could be that, given the concerns raised in previous sections, neither condition provided an accurate sample from the truly concrete or abstract sections of the scale. In the second experiment that I will report, the standard deviations of the conditions were more tightly constrained so that in the concrete and abstract conditions, all standard deviations were below 1.

## Experiment 2

Paivio, Walsh, and Bons (1994) presented participants with lists consisting of both concrete and abstract word pairs and reported that concrete word pairs were recalled better than abstract word pairs. This effect has been obtained in many paired-associate learning experiments (Begg, 1972; Nelson & Schreiber, 1992; Paivio, Khan, & Begg, 2000; Paivio et al., 1994). Paivio et al. employed a range of different manipulations across two experiments. In this replication I focused on the simplest version of this paradigm, which is a free-recall task, in order to make the results maximally comparable to those of Experiment 1 above. The aim of the present experiment was to test whether a concreteness effect still occurs if the contrast between concrete and abstract stimuli is maximized and the standard deviations of their concreteness scores are controlled. In addition to the concrete and abstract conditions featured in the paired-associate learning studies mentioned in this section, the present experiment also included a midscale condition to provide a second test of the hypothesis that high-standard-deviation midscale words are harder to remember than words from the concrete end of the concreteness scale.

## Method

### Participants

Sixty native speakers of English with no reported neurological disorders were recruited from the Prolific Academic website. All participants were paid £6 for their time.

### Materials

**Fig. 8:**
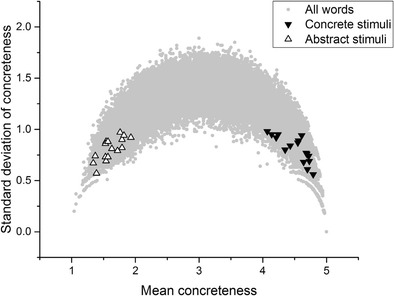
Concrete and abstract stimuli featured in Experiment 2

Figure 8 depicts the means and standard deviations of the concreteness ratings for the concrete and abstract stimuli in Experiment 2. Table 6 displays the psycholinguistic characteristics of the stimuli featured in the experiment, by condition.

**Table 6:** Summary of stimulus characteristics for Experiment 2

Condition	Mean Concreteness	*SD* Concreteness	AOA	Zipf Frequency	L Phon	*N* Syll	Length	BG Mean
Concrete	4.51 (0.23)	0.91 (0.13)	9.92 (1.9)	3.54 (0.56)	4.75 (0.2)	1.75 (0.43)	6.125 (1.41)	3,573 (1,151)
Abstract	1.61 (0.17)	0.81 (0.11)	10.04 (1.64)	3.48 (0.69)	5.25 (1.44)	1.75 (0.43)	6.44 (1.5)	3,457 (1,176)
Disagreement	3 (0.23)	1.33 (0.02)	9.78 (1.95)	3.72 (0.78)	5.75 (1.48)	1.81 (0.39)	6.38 (1.45)	3,218 (957)

Mean concreteness: Mean concreteness rating; *SD* concreteness: The mean standard deviation of the concreteness ratings; AoA: Age of acquisition; Zipf frequency: Word frequency in Zipf units; L Phon: Length of word in phonemes; *N* Syll: Number of syllables; Length: Length of word in letters; BG mean: Mean bigram frequency

In Experiment 2 the additional control variable of mean bigram frequency was introduced, because participants would be reading and writing words as opposed to hearing and speaking them. There were eight pairs of words in each condition, and therefore each condition included 16 words, for a total of 24 critical item pairs overall.

### Procedure

Participants undertook the experiment online via a Qualtrics survey distributed over the Prolific Academic service. Participants were presented with pairs of words, one after the other. Following Marschark and Hunt (1989) and Paivio et al. (1994), each pair of words was presented on the participant’s computer screen for 8 s. Eight pairs were presented in each of the three conditions, and all pairs were presented in a randomized nonblocked order for each participant. The ordering of the words in each pair from left to right on the computer screen was not randomized. At the beginning and end of the list, three pairs of filler items were included in order to soak up primacy and recency effects. Participants also received a short practice trial with words not included in the main experiment, to ensure that they understood the task and that their computers and Internet connections were working properly. Once the list of pairs was finished, participants could type out any and all words that they remembered from the list. Once they were finished, they pressed a “Submit” button that ended the experiment. There were three experimental conditions: A word pair could consist of concrete, abstract, or midscale “disagreement” items. The experiment lasted approximately 15 min.

## Results

Table 7 displays the mean numbers of words remembered across conditions in Experiment 2.

**Table 7:** Mean words recalled by condition in Experiment 2

Condition	Mean Words Recalled	Mean Percentage Recalled
Concrete	3 (2.73)	18.6%
Abstract	3.43 (3.07)	21.5%
Disagree	3.05 (2.84)	19.1%

The numbers of words recalled out of 16 were low, but the variability across participants was large, as indicated by the relatively high standard deviations of the mean numbers of words recalled. This suggests floor effects for some participants. Second, the mean number of words in the abstract condition was numerically larger than that in the concrete condition (3 < 3.43), so already we have failed to find evidence in favor of a concrete stimulus advantage in paired-associate learning. Finally, the difference between the means of the concrete and disagree conditions was miniscule (3 vs. 3.05, respectively).

The data were analyzed using a generalized linear mixed model fit by maximum likelihood (Laplace approximation) using the glmer function from the lme4 package in R. The dependent variable in this analysis was therefore the likelihood of a participant recalling any word.^1^ Subjects and items were included as random effects with varying intercepts, and the fixed effect of condition was the effect of interest. Both abstract and disagree conditions were compared to the concrete condition. The results of this analysis are presented in Table 8.

**Table 8:** Summary of a generalized linear mixed model analysis of Experiment 2

Effect	Effect Estimate	Std. Error	*z*	*p*
Abstract	.19	.15	1.3	.2
Disagree	.02	.15	.15	.88

Experiment 2 generated no statistically significant effects: *p* = .2 for the concrete–abstract contrast, and *p* = .88 for the concrete–disagree contrast. This pattern of results is the same as that found in Experiment 1: Under conditions that should have made a concreteness effect stronger, such an effect was not obtained. However, ultimately we should be cautious in drawing any conclusions from the results of Experiment 2, because floor effects may have obscured any differences between conditions.

## Interim summary

Experiments 1 and 2 did not produce a concreteness effect. This is worrying, given the concerns about the typically high standard deviations of abstract stimuli outlined above. If we increased a difference between conditions on some linear measure, we would not expect experimental effects based on this measure to disappear. However, Kousta, Vinson, and Vigliocco (2009) showed that words with a high emotional valence (whether positive or negative) enjoy a processing advantage over words with neutral emotional valance.^2^ Abstract words tend to be rated higher for emotional valance than concrete words, and this variable was not controlled in Experiment 1 or 2. Thus, it could be that a confound in the stimuli used in Experiments 1 and 2 obscured any concreteness effect. Warriner et al.’s (2013) emotional valance norms for ~14,000 English words would allow us to check this possibility. Emotional valance is rated on a scale of 1 (*highly negative*) to 9 (*highly positive*), with a score of 5 indicating an emotionally neutral word. Given that either emotional positivity or negativity results in a processing advantage, the absolute value of 5 minus the emotional valance of a word provides a simple linear measure of emotional valance that ignores polarity (0 = *totally neutral*, 4 = *highly emotionally valenced*). Table 9 presents the mean absolute emotional valences of the stimuli featured in Experiments 1 and 2.

**Table 9:** Emotional valences of stimuli featured in Experiments 1 and 2

Experiment	Concrete	Abstract	Disagree	Agree
1	0.82	1.17	0.88	1.15
2	0.91	1.61	0.99	N/A

The words in the concrete and midscale conditions were indeed less emotionally valenced than those in the abstract conditions in both experiments, so this might explain the null results obtained from Experiments 1 and 2.

Another potential issue is that the words featured in Experiments 1 and 2 were of relatively low frequency (between 3 and 4 on the Zipf scale), so it could be that participants did not know all of the words.^3^ This could have obscured any effect of manipulating concreteness. Brysbaert et al. (2013) provided a measure of how many of their participants reported that they knew a word. Table 10 below displays the mean percentages of participants who reported knowing a word for each condition in Experiments 1 and 2.

**Table 10:** Mean percentages of participants who reported in Brysbaert et al. (2013) knowing the words featured in Experiments 1 and 2

Experiment	Concrete	Abstract	Disagree	Agree
1	98.5%	98.3%	97.7%	98.5%
2	99.5%	99.1%	98%	N/A

These percentages are high, so it is likely that the number of participants in Experiments 1 and 2 who did not know a word was very low. However, it would obviously be preferable if only words with known percentages of 100% were used. Unfortunately, for reasons detailed in the General Discussion below, enforcing this control raised new problems. I now report an additional list memory experiment that controlled for emotional valence, in order to provide a better test of the robustness of the concreteness effect.

## Experiment 3

Experiment 3 was a free-recall list memory experiment in the vein of Experiment 1. There were three changes to the paradigm. First, six-word lists were used instead of eight-word lists. This change was made so that more trials per condition (15 in Exp. 3 vs. 10 in Exp. 1) could be fitted into roughly the same amount of time. Romani et al. (2008) and Miller and Roodenrys (2009) both reported concreteness effects with six-word lists. Second, the words were presented visually, and participants wrote out the words at the end of a list instead of speaking them out loud. This change was made because to maximize efficiency, the experiment was run over the Internet using the Gorilla.sc platform. Finally, only three conditions were included: concrete, abstract, and midscale words with high standard deviations.

## Method

### Participants

A total of 70 participants were recruited from the Prolific Academic website. Of these, 62 completed the experiment. The other eight did not respond to every trial, and so were excluded. The experiment was delivered via Gorilla.sc and lasted approximately 35 min. Participants were paid £5 for their time.

### Materials

The stimuli were controlled for the following psycholinguistic variables: standard deviation of the concreteness rating, frequency, age of acquisition, number of syllables, number of letters, mean bigram frequency, and emotional valence. Table 11 contains the mean values (with standard deviations in parentheses) of each of these variables for each condition, as well as the mean percentages of people in the Brysbaert et al. (2013) norms who reported knowing the words in each condition.

**Table 11:** Summary of stimulus characteristics for Experiment 3

Condition	Mean Concreteness	*SD* Concreteness	AoA	Zipf Frequency	*N* Syll	Length	BG mean	Absolute Valence	Percent Known
Concrete	4.55 (0.17)	0.81 (0.12)	10.11 (1.28)	3.41 (0.48)	2.42 (0.86)	7.63 (1.79)	3,649 (1,134)	1.12 (0.77)	99%
Abstract	1.61 (0.15)	0.85 (0.11)	10.2 (1.95)	3.54 (0.72)	2.53 (0.89)	7.63 (1.95)	3,710 (1,208)	1.15 (0.78)	99%
Midscale	3.02 (0.26)	1.51 (0.77)	10.11 (1.99)	3.53 (0.72)	2.54 (0.86)	7.57 (1.89)	3,737 (1,184)	1.15 (0.77)	98.7%

Mean concreteness: Mean concreteness rating; *SD* concreteness: The mean standard deviation of the concreteness ratings; AoA: Age of acquisition; Zipf frequency: Word frequency in Zipf units; *N* Syll: Number of syllables; Length: Length of word in letters; BG mean : Mean bigram frequency; Absolute Valence: Absolute value of 5 minus the Warriner et al. (2013) emotional valence score.

There were three experimental conditions: concrete, abstract, and midscale. There were 15 six-word lists in each condition.

### Procedure

Participants were presented with words in sequence one at a time in the center of their computer screens. As in Romani et al.’s (2008) visual paradigms, each word remained on the screen for 3 s. After each list had been presented, participants typed out any and all words that they could remember. They were told that the order of the words did not matter and not to worry about spelling. Participants received two practice trials in order to ensure that they understood how to complete the experiment. The orders of the lists and of the words within each list were randomized for each participant.

## Results

Table 12 summarizes the mean numbers of words remembered (and standard deviations) by condition.

**Table 12:** Mean words recalled by condition for Experiment 3

Condition	Mean Words Recalled (*SD*)	Mean Percentage Recalled
Concrete	4.06 (1.31)	67.7%
Abstract	3.7 (1.25)	61.7%
Midscale	3.85 (1.28)	64.2%

The results from Experiment 3 were analyzed in the same way as the results from Experiment 1. Both frequentist and Bayesian analyses are presented. Table 13 displays the results of a mixed-effects linear model with a fixed effect of condition and random intercepts for subjects and items.

**Table 13:** Summary of frequentist mixed-effects model for Experiment 3

Fixed Effects	Effect Estimate	Error	*df*	*t*	*p*	Lower 95% CI for Effect	Higher 95% CI for Effect
Abstract	–.37	.12	44.34	–3.11	.003	–.61	–.13
Midscale	–.21	.12	44.34	–1.79	.08	–.45	.03

After controlling for the effects of emotional valence, these results are much more encouraging for the status of concreteness as a useful psycholinguistic variable. The concrete–abstract comparison is statistically significant at *p* = .003, and the difference is in the direction we would expect. The contrast between the concrete and midscale conditions was not statistically significant (*p* = .08). Because this experiment still featured a relatively small number of items, a Bayesian model comparison analysis was deployed in an attempt to offset a potential lack of power. Again, the default settings and priors of the BayesFactor package were used. As in Experiment 1, the results from Experiment 3 were split into subsets so that the abstract and midscale conditions would be compared to the concrete condition individually. The resulting Bayes factors for each comparison were concrete versus abstract, 5.85; concrete versus midscale, 0.47. For the concrete–abstract comparison, the Bayesian analysis is comparable to the frequentist analysis: A model containing an effect of condition is 5.85 times more likely given the data than a model without this effect, which is quite strong evidence in favor of a concreteness effect. However, the concrete–midscale analysis was inconclusive. One thing to note is that Experiment 3 featured words with similar rates of knowledge to those in Experiments 1 and 2. Experiment 3 produced a concreteness effect, so this might partially allay concerns that Experiments 1 and 2 produced null results because participants did not know the words used. I now turn to a general discussion of these results in light of the issues discussed in the introductory section on concreteness norms, as well as a consideration of other psycholinguistic variables (imageability, modality exclusivity norms, and emotional valence).

## General discussion

The first two experiments did not produce a concreteness effect, but these experiments featured a confound: The abstract stimuli had higher emotion ratings than the concrete stimuli. Experiment 3 controlled for emotional valence, and the typical concreteness effect reemerged. This highlights the importance of controlling for emotional valence in list memory paradigms. There were no statistically significant differences between the concrete conditions and the midscale conditions in any experiment. This demonstrates that researchers who are interested in the concreteness effect should maximize the contrast between concrete and abstract stimuli and keep the standard deviations of their stimuli low (below 1) in order to maximize their chances of detecting an effect.

It might seem curious that, given that other list memory studies have revealed concreteness effects when comparing mostly concrete stimuli with mostly high-standard-deviation midscale stimuli, no such effect was obtained in any of the experiments reported here. As I argued when discussing the Brysbaert et al. (2013) norms, the middle of the concreteness scale is marked by a high degree of variability that is difficult to interpret. One of the aims of this article was to test the possibility that words that people agree about how to rate are easier to remember than words that people disagree about how to rate. The three experiments reported here do not provide evidence either way on this point: *p* values above .05 (corrected) and Bayes factors between 1/3 and 3 for the concrete–midscale comparisons indicate evidence for neither the null nor the alternative hypothesis. The most likely reason for this is a lack of power: The experiments presented here did not feature many stimuli per condition. However, as I will discuss below, this problem is harder to address than might first appear. Furthermore, the abstract conditions in the experiments of Romani et al. (2008), Walker and Hulme (1999), Miller and Roodenrys (2009), and Allen and Hulme (2006) were not *entirely* made up of midscale stimuli. So if there is a concreteness effect in list memory experiments, the abstract–concrete comparisons in these previous experiments would be more likely to detect it than were the concrete–midscale comparisons reported here.

This issue aside, in light of my arguments regarding Brysbaert et al. (2013), we should probably avoid using midscale words on purely theoretical grounds: It is unclear what an individual concreteness rating is even measuring when it has a high standard deviation. A reviewer raised the point that abstract words tend to have more variable meanings than concrete words, so more variability in their ratings might be expected. This may be true, but I think it somewhat misses the point. If there is any point in using the concreteness measure (or the other measures I discuss below), we have to take our participants’ ratings seriously. If a word in the middle of the scale has a standard deviation above 1, that means a significant number of participants judged it to be concrete. Thus, there isn’t a basis for putting that word in the “abstract” category: It does not make sense to pay attention to only half of the participants’ judgments. There is another potential issue, even if we make sure to restrict our “abstract” stimuli to mean ratings of 2 or below. Typically, concreteness research has focused on nouns rather than adjectives or verbs. Even starting with a set of 40,000 words, the number of nouns in the Brysbaert et al. (2013) norms that (1) have a mean rating of 2 or below (i.e., are highly abstract), (2) have a standard deviation of 1 or below, and (3) were known by 100% of the norming population is only 275. Of these, a small but nontrivial number are either idiomatic fragments (“amuck”) or morphologically complex rarities (“purposefulness”) that we might be reluctant to include in stimulus lists. In contrast, 2,888 well-known nouns have mean ratings of 4 or above and standard deviations below 1.

I think this fact should also motivate caution concerning the utility of the concreteness measure. Ultimately, the measure is supposed to tap into a fundamental, neuropsychologically real distinction between different kinds of concepts. It is worrying that the rating is only interpretable for a small number of nominal “concepts” at the abstract pole. However, it is still the case that Experiment 3 produced a concreteness effect. At the very least, we can say that there is some evidence that samples of these “truly” abstract words tend to be harder to remember than highly concrete words.

**Fig. 9:**
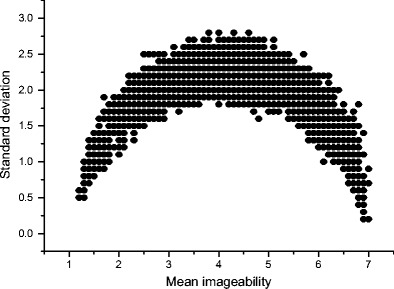
Means and standard deviations of imageability ratings for 6,000 words (Cortese & Fugett, 2004; Schock et al., 2012)

I turn now to a discussion of other semantic psycholinguistic variables. The midscale variability problem applies to other variables that measure sensorimotor experience. This is not surprising, because these variables are derived in much the same way as concreteness (by taking the mean value of a set of individual judgments about the depth of sensorimotor experience). This is especially significant because it shows that nothing about the Brysbaert et al. (2013) concreteness norms is deficient. Instead, the problems I have identified here are general to a whole class of psycholinguistic measures. Figure 9 presents a mean–standard deviation plot of the imageability ratings of 6,000 words, amalgamated from two databases (Cortese & Fugett, 2004; Schock et al., 2012). Imageability is a measure of how easy it is to generate a mental image of the referent of a word, and this variable is so highly correlated with concreteness that the two have often been used interchangeably in the literature.

**Fig. 10:**
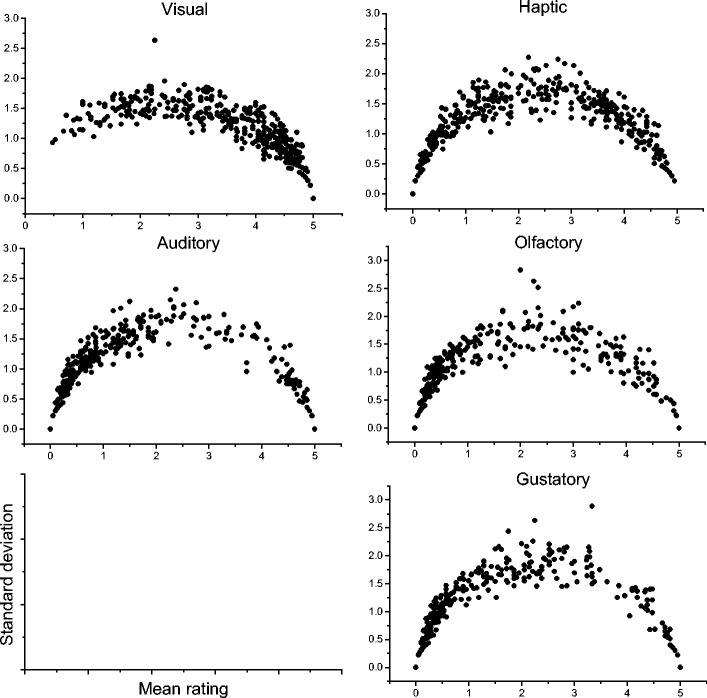
Means and standard deviations of Lynott and Connell’s (2012) modality exclusivity norms

The distribution is identical to that of the concreteness measure. A similar pattern emerges for Lynott and Connell’s (2012) modality exclusivity norm (MEN). MEN essentially measures the same thing as concreteness, but it provides more information because it features ratings for all five primary sensory modalities (sight, sound, touch, taste, and smell). A low rating indicates that the referent of a word offers little experience in a given modality; a high rating indicates that a referent offers a lot of experience. Each word is rated on all five modalities. This results in a five-element vector from which various measures can be derived (mean sensory experience, maximum sensory experience, Euclidean distance from origin, etc.). Figure 10 displays mean–standard deviation plots of all 400 words in the MEN for the five sensory modalities.

What is striking here is that even with just 400 words, the familiar shape of the distribution is clearly apparent. I do not think that we can ignore the fact that all of these datasets have the same problematic distribution. This is likely to be a result of the question that we ask participants when we generate these measures. When we present depth of sensorimotor experience as a scale, we are implicitly committing to the idea that is possible for an entity to be “half-real,” or “half in space–time,” or “half-seeable.” The distributions of these semantic variables tell us that participants tend to reject this idea: They do not use midscale values.

One solution might be to specify explicitly what we want the middle of these scales to represent, and to provide examples of midscale words for participants so that they have something to anchor their judgments to. Whether something along these lines would usefully decrease variability in the middle of the scale is an open question, but a potential issue here is that it is very difficult (for me) to think of a construct that could serve as a midscale anchor between “concreteness” and “abstractness.” More worryingly, given that there are relatively few words in the abstract half of the scale with low standard deviations, it could be that the concrete–abstract dichotomy is just not well formed.

**Fig. 11:**
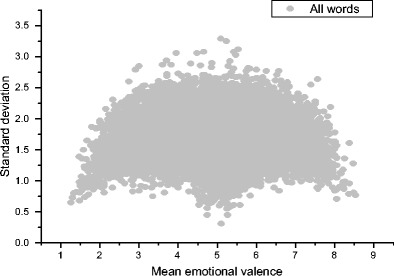
Means and standard deviations of Warriner et al.’s (2013) emotional valence norms

Finally, I want to briefly discuss the distribution of emotional valence ratings. Emotional valence is different from the sensorimotor variables discussed above, in that it measures a completely separate dimension of experience. The standard deviation of an emotional valence rating also takes on a special importance because of how the scale is constructed. Figure 11 presents the means and standard deviations of the emotional valence scores from Warriner et al. (2013) (*n* = 13,900). Warriner et al. presented this plot and touched on this issue, but they did not raise exactly the same point as the one I want to focus on here.

Recall that a score of 1 indicates *extremely negative emotional valence*, 5 indicates *neutrality*, and 9 indicates *extremely positive emotional valence*. Looking at Fig. 11, it should be possible to select unequivocally negative, neutral, and positive words for use in experiments: There are some words at mean ratings of 1, 5, and 9 with low standard deviations. This is obviously a good thing.

However, because the middle of this scale is a neutral point between two extremes, words with high standard deviations are especially problematic. This is because a 5 is supposed to indicate emotional neutrality. But if a word has a mean of 4–6 but a standard deviation of 2 or more, that means that on average, participants actually associate moderate to large emotional responses with that word. Some participants associate positive emotions with the word, but others associate negative emotions with it. Quite a few words *look* neutral, but in fact are not. A few examples are:

- *Cell*: Mean = 4.09, *SD* = 2.69
- *Sushi*: Mean = 6.25, *SD* = 2.77
- *Gym*: Mean = 5.84, *SD* = 2.52

Similarly, if a word has a mean emotional valence of, say, 3, but a standard deviation above 1.5, that means that some people report a very strong negative response to that word, whereas some people report little or no emotional response at all. So if a researcher is interested in comparing responses to neutral words with responses to emotionally valenced words, they should definitely avoid words with high standard deviations for emotional valence, because they will add a significant amount of noise to the experimental design. One positive thing to note is that for the emotional valence measure, a high standard deviation is potentially problematic but is still *interpretable.* It makes sense that different people will associate different emotions with certain words. It also makes sense to think of our emotional responses as graded. I think this is a key difference between the sensorimotor experience variables and the emotional valence measure.

## Conclusion

I have argued that there is a problem with the statistical characteristics of various semantic psycholinguistic variables (focusing in particular on the concreteness variable). In a great number of cases, mean values do not reflect the judgments that actual participants made about a word. Furthermore, mean values in the middle of these scales are difficult to interpret because it is not clear what property they indicate. Unfortunately, it appears that in many experiments reported throughout the literature on concreteness effects, many of the stimuli in the abstract conditions are not actually abstract. Instead, they are precisely those stimuli for which the mean concreteness value is a bad indicator of what participants’ choices were. In two of the new list memory experiments reported here, no concreteness effect was obtained when the contrast in concreteness between conditions was maximized. However, when emotional valence was controlled, a concreteness effect was obtained in Experiment 3.

The concreteness effect obtained in Experiment 3 is encouraging, because it allays some of the concerns outlined above. However, there are still a number of reasons to be cautious about concreteness and other related semantic variables. First, the status of words with high standard deviations is entirely unclear. These high standard deviations for midscale words might arise at least partially because it is unintuitive to treat sensorimotor experience as a graded property. Second, only a very small number of “abstract” nouns have low standard deviations. This calls into question the utility of the concreteness–abstractness dichotomy as it is currently operationalized. Also, researchers who want to use nominal stimuli or control for word class have very little choice if they want to keep the standard deviations of their stimuli low. For the emotional valence measure, I think the picture is somewhat better. High standard deviations provide meaningful information, although it is perhaps even more important to keep standard deviations low when making comparisons between different areas of the scale.

The good news is that the use of new large-scale psycholinguistic databases such as the Brysbaert et al. (2013) concreteness norms and Warriner et al.’s (2013) emotional valence norms rather than relatively small, older databases (Coltheart, 1981) can allow researchers to sidestep the problems I raise completely. This is because the sheer size of these datasets allows for the selection of suitable stimuli.

## Appendices

**Table 14:** 1. Experiment 1 stimuli

**List**	**condition**	**Word 1**	**Word 2**	**Word 3**	**Word 4**	**Word 5**	**Word 6**	**Word 7**	**Word 8**
1	**disagree**	polling	dipstick	decade	centaur	exhaust	foreword	limbo	spender
2	**disagree**	physic	sequel	deacon	nettle	output	earshot	deadline	cackle
3	**disagree**	brethren	zenith	deluge	silence	lawsuit	theorist	polka	margin
4	**disagree**	nappy	degree	panic	bearings	legend	request	physics	prefect
5	**disagree**	sponsor	delta	dropper	phantom	egghead	rightness	aerial	eyesight
6	**disagree**	halter	brainwave	mankind	nightlife	surname	scrounger	tunic	omen
7	**disagree**	pariah	divorce	cosmos	sundries	purveyor	demon	crosswind	alias
8	**disagree**	grammar	conveyance	easement	blackball	woodland	giantess	weeknight	instant
9	**disagree**	tidbit	shallows	photon	plural	hallmark	grafting	sandman	nature
10	**disagree**	slipstream	audit	poorhouse	minute	rival	tribune	abyss	spectrum
11	**agree**	menace	bookie	tinting	flicker	rebound	squatter	tempo	pusher
12	**agree**	uprise	digest	tiling	region	charmer	joyride	outbreak	nutrient
13	**agree**	hubbub	matron	median	nuthouse	pullout	partner	distaste	refill
14	**agree**	burial	backwash	mover	career	event	footing	caper	peacetime
15	**agree**	jailbreak	torment	hazard	instinct	guru	downpour	richness	glucose
16	**agree**	bunting	rhythm	stalker	dullness	ascent	headache	gunpoint	welfare
17	**agree**	ringside	archduke	turmoil	shyness	posse	gangway	shipping	outreach
18	**agree**	sunburst	mishap	bumpkin	deceit	villain	bloodlust	misdeed	hunting
19	**agree**	diesel	roughhouse	attempt	whiner	viewpoint	freshness	stampede	leader
20	**agree**	semblance	havoc	broadside	dining	image	dissent	goner	culprit
21	**abstract**	setback	vagueness	spirit	notion	loyalty	esteem	phrasing	credence
22	**abstract**	charade	rapture	betrayal	logic	backlash	renown	letdown	affront
23	**abstract**	desire	mystique	intent	vantage	glory	nuance	unease	motive
24	**abstract**	amends	prestige	godsend	satire	leeway	wordplay	pretense	calmness
25	**abstract**	accord	whimsy	disdain	hardship	virtue	manner	regard	effect
26	**abstract**	freelance	mischief	respite	folly	pureness	repute	courage	meantime
27	**abstract**	merit	standpoint	future	allure	rapport	wisdom	prudence	insight
28	**abstract**	mistake	quantum	dogma	function	purpose	willpower	hearsay	meaning
29	**abstract**	patience	aspect	debut	fairness	pity	taboo	riddance	appeal
30	**abstract**	piety	finesse	foresight	longshot	loathing	stigma	concern	control
31	**concrete**	leaflet	roadhouse	artist	lighting	parsley	seabed	ironwork	lacrosse
32	**concrete**	clipper	pewter	cauldron	quarry	blockade	earwig	clubfoot	logbook
33	**concrete**	summit	breeches	abscess	foreman	award	entree	funnel	beacon
34	**concrete**	corset	template	pigment	fuchsia	urchin	ringworm	crewman	mansion
35	**concrete**	jester	gasket	sternum	backdrop	bouncer	chapel	resort	county
36	**concrete**	penthouse	fracture	entrails	vinyl	buckskin	tundra	barrier	plumbing
37	**concrete**	timepiece	methane	record	tiller	grindstone	merchant	shrapnel	duchess
38	**concrete**	quarter	bulkhead	sarong	tenant	chamber	canon	bailiff	machine
39	**concrete**	beaker	clinic	tango	clothing	amber	jackal	roulette	survey
40	**concrete**	spiral	marrow	billiard	bootlace	scabies	saffron	captain	product

**Table 15:** 2. Experiment 2 stimuli

**Pair**	**Condition**	**Word 1**	**Word 2**
1	**concrete**	cauldron	hike
2	**concrete**	footman	band
3	**concrete**	blazer	creature
4	**concrete**	rubble	liqueur
5	**concrete**	throttle	ulcer
6	**concrete**	ranch	gauntlet
7	**concrete**	cadet	concert
8	**concrete**	ledge	manor
9	**abstract**	betrayal	urge
10	**abstract**	revenge	foresight
11	**abstract**	godsend	risk
12	**abstract**	wisdom	psyche
13	**abstract**	hardship	malice
14	**abstract**	greed	riddance
15	**abstract**	loyalty	lenience
16	**abstract**	bliss	mercy
17	**midscale**	genius	royalty
18	**midscale**	foreground	district
19	**midscale**	gleam	patriot
20	**midscale**	view	approach
21	**midscale**	upstart	brawn
22	**midscale**	expanse	profit
23	**midscale**	asset	vortex
24	**midscale**	habit	encore

**Table 16:** 3. Experiment 3 stimuli

**List Number**	**Condition**	**Word 1**	**Word 2**	**Word 3**	**Word 4**	**Word 5**	**Word 6**
1	concrete	pad	harpoon	stretcher	kennel	ulcer	aftershave
2	concrete	trachea	parsley	fuselage	rifleman	plaster	medallion
3	concrete	cedar	rubble	trinket	composer	liver	dormitory
4	concrete	scale	shipment	gladiator	guesthouse	morgue	marrow
5	concrete	vineyard	porcelain	cocktail	warship	advisor	slate
6	concrete	supervisor	infirmary	bouquet	manicure	bay	tomb
7	concrete	graphics	sage	smoothie	wildfire	prosecutor	sapphire
8	concrete	inspector	minefield	tourist	stub	horseradish	frostbite
9	concrete	guitarist	notch	gauntlet	orphanage	vegetation	bomber
10	concrete	greenhouse	sedative	museum	silicon	wreckage	accountant
11	concrete	incubator	lavender	surgeon	violinist	courtroom	embroidery
12	concrete	landlord	measles	dictator	pacemaker	minibus	plumber
13	concrete	newsletter	bodyguard	stockbroker	foliage	petroleum	liqueur
14	concrete	plantation	attorney	blockade	antibiotic	concert	currency
15	concrete	stroke	titanium	bile	sniper	massage	adhesive
16	abstract	urge	renown	patience	motive	malice	quandary
17	abstract	penance	belief	indulgence	reproach	version	fixation
18	abstract	mercy	glory	charade	aptitude	manner	formality
19	abstract	risk	psyche	rhetoric	foresight	fraud	regard
20	abstract	prudence	oblivion	hardship	mood	sarcasm	fate
21	abstract	extent	imposition	purpose	competence	luck	whim
22	abstract	willpower	bias	indecision	loyalty	seriousness	knowledge
23	abstract	involvement	existence	coincidence	ruse	principles	betrayal
24	abstract	detriment	subtlety	tradition	damnation	wisdom	fantasy
25	abstract	forgiveness	semantics	value	sanctity	godsend	discretion
26	abstract	eternity	politeness	concept	reasoning	anomaly	symbolism
27	abstract	suspicion	goodness	arrogance	mortality	chance	theory
28	abstract	precedent	privacy	likelihood	lunacy	oversight	revenge
29	abstract	affirmative	repentance	leniency	similarity	merit	expertise
30	abstract	wickedness	analogy	bliss	coercion	courage	avoidance
31	midscale	plot	molecule	mankind	format	swindle	motherland
32	midscale	hormone	reply	tarot	tribune	routine	pushover
33	midscale	delay	gossip	slumber	bandwagon	response	vigilante
34	midscale	zone	shallows	pinnacle	wavelength	grief	degree
35	midscale	envoy	character	fallout	clue	vacancy	tone
36	midscale	circulation	drunkenness	midsummer	doctorate	goal	hoax
37	midscale	cutthroat	rift	corporation	lawsuit	translation	sweetness
38	midscale	announcement	activist	process	slack	formation	whiplash
39	midscale	chronicle	monologue	overlap	motherhood	virus	penalty
40	midscale	exhaustion	delegate	magic	rebuttal	crackpot	diversion
41	midscale	entirety	ugliness	factor	ancestry	confidant	purgatory
42	midscale	engagement	accident	insomnia	regulator	utility	egghead
43	midscale	repellent	takeover	provision	dioxide	offence	thinker
44	midscale	equivalent	oracle	ignition	visibility	ransom	narrative
45	midscale	sense	extremity	content	lunatic	divorce	casualty

## Footnotes

1 A reviewer noted that analyzing the data in this way meant that this experiment was arguably no longer a paired-associate learning task, presumably because it did not account for the paired relationship between the words. In their free-recall analyses, Paivio et al. (1994) calculated the proportions of words remembered and conduct by-subjects and by-items ANOVAs on these proportions. These analyses also ignored word pair relationships and produced concreteness effects, so I think we would still expect the analysis presented here to produce a concreteness effect.

2 My thanks to an anonymous reviewer for bringing this to my attention.

3 Again, my thanks to an anonymous reviewer for pointing this out.
